# Iron Oxide Nanoparticles in Bioimaging – An Immune Perspective

**DOI:** 10.3389/fimmu.2021.688927

**Published:** 2021-06-15

**Authors:** Mark Geppert, Martin Himly

**Affiliations:** Division of Allergy and Immunology, Department of Biosciences, University of Salzburg, Salzburg, Austria

**Keywords:** SPIONs, inflammation, oxidative stress, ROS, MRI contrast agent, endotoxin

## Abstract

Iron oxide nanoparticles (IONPs) bear big hopes in nanomedicine due to their (potential) applications in tumor therapy, drug delivery or bioimaging. However, as foreign entities, such particles may be recognized by the immune system and, thus, lead to inflammation, hypersensitivity or anaphylactic shock. In addition, an overload with iron is known to cause oxidative stress. In this short review, we summarize the biological effects of such particles with a major focus on IONP-formulations used for bioimaging purposes and their effects on the human immune system. We conclude that especially the characteristics of the particles (size, shape, surface charge, coating, *etc.*) as well as the presence of bystander substances, such as bacterial endotoxin are important factors determining the resulting biological and immunological effects of IONPs. Further studies are needed in order to establish clear structure-activity relationships.

## Introduction

Nanoparticles (NPs) are defined as particular entities with a maximum size of 100 nm in two or three dimensions (1). They can be composed of different organic and inorganic materials such as liposomes, micelles, carbon nanotubes, fullerenes, metalloids, metals and metal oxides. Iron oxide nanoparticles (IONPs) typically consist of a core of a magnetic iron oxide (magnetite, Fe_3_O_4_ or maghemite, γ-Fe_2_O_3_), surrounded by a coating for their stabilization and can be synthesized by a huge variety of chemical approaches (2, 3). During the last decades, IONPs became more and more popular due to their numerous biomedical applications, such as cancer therapy by magnetic-field mediated hyperthermia, drug-delivery, iron replacement therapy or bioimaging techniques (4–6). Although IONPs are generally considered as biocompatible and possess only low cytotoxic potential (7), it is evident that such particles are considered as foreign entities and, thus, may have a variety of effects on the immune system, such as hypersensitivity reactions, immunosuppression or immune stimulation (8, 9). The aim of this review is to give an overview on different IONP formulations that are (or have been) clinically applied for imaging purposes and to elaborate on the effects of these particles on human health and the immune system.

## Iron Oxide Nanoparticles in Bioimaging

Due to the magnetic properties of the core and the small particle size, IONPs are superparamagnetic and undergo magnetization only in presence of a magnetic field. This special properties have shown to be beneficial for use of such particles as contrast agents in magnetic resonance imaging (MRI) where IONPs produce hypointense (dark) signals in T_2_-weighted MR images (10, 11). As such, they represent the counterpart to the classical Gadolinium (Gd)-based contrast agents which increase T_1_ relaxation rates leading to positive (bright) image contrast (12). In recent literature, there is also evidence that ultra-small IONPs (<5 nm) can be used in both T_1_- and T_2_-weighted MRI (13, 14). These findings could appear to be beneficial in the future since Gd-based contrast agents are known to be less biocompatible and can raise the possibility of nephrotoxicity, especially in patients with advanced acute or chronic kidney disease (15, 16).

MRI contrast agents can be administered orally or intravenously (17). An example for an orally administered IONP-based contrast agent is Ferumoxsil (Lumirem^®^, GastroMARK^®^), which was approved for imaging of the gastrointestinal (GI) tract by the FDA in 1996 but later withdrawn from the market. However, most IONP-based imaging agents are administered *via* injection. Ferumoxide (Feridex^®^, Endorem^®^) and Ferucarbotran (Ciavist™, Resovist^®^) were two contrast agents designed for liver imaging which both became approved in the US as well as in Europe. However, due to different side effects, both were withdrawn from the market in 2008/2009 and only Resovist^®^ is still available in limited countries (9, 18, 19). Ferumoxtran-10 (Sinerem^®^, Combidex^®^) was designed for lymph node imaging in prostate cancer metastasis (20). However, also this earlier FDA-approved agent was withdrawn from the market due to a lack of improved efficacy (19). Ferumoxytol (Feraheme^®^, Rienso^®^) was initially approved for treatment of iron deficiency for patients with chronic kidney disease but recently has also been shown to be suitable as contrast aging in MRI, however with no clinical approval yet (21). VSOP C184 and Feruglose (Clariscan^®^) were both designed and clinically tested for MR angiography and blood pool imaging but did not reach the level of approval (22, 23). **Table 1** lists a selection of iron oxide-based contrast agents applied for bioimaging.

**Table 1:** Selected IONP-based contrast agents for MRI.

Formulation	Trade name(s)	Coating	Application(s)
Ferumoxide	Feridex^®^, Endorem^®^	Dextran	Liver imaging
Ferucarbotran	Ciavist™, Resovist^®^	Carboxydextran	Liver imaging
Ferumoxtran-10	Sinerem^®^, Combidex^®^	Dextran	Lymph node imaging
Ferumoxytol	Feraheme^®^, Rienso^®^	Polyglucose sorbitol carboxymethyl ether	CNS imaging, blood pool imaging, lymph node imaging, Iron deficiency treatment
Citrate-coated very small iron oxide NP	VSOP C184	Citrate	MR angiography, Blood pool imaging
Feruglose	Clariscan^®^	PEGylated starch	MR angiography, Blood pool imaging
Ferumoxsil	Lumirem^®^, GastroMARK^®^	Siloxane	Oral GI imaging

Aside from MRI, IONPs can also be applied for a similar but different technique, named magnetic particle imaging (MPI). Here, the particles do not act as a contrast agent but rather are the only source of the signal and the only visualized element (24). By using static and dynamic magnetic fields, the unique magnetic characteristics of IONPs can be used to acquire detailed images without using harmful ionizing radiation. In contrast to MRI, MPI thereby only images the anatomical sections which were labelled with the IONP tracer (25, 26). Thereby it offers three-dimensional images with high sensitivity, temporal and spatial resolution. However, to date, MPI is rather experimental, and available scanners are designed for investigation of mouse- and rat-sized animals (26). IONP-formulations like Ferucarbotran (Resovist^®^) have been shown suitable for MPI, however, other formulations of IONPs (IONP-micelles) seemed to perform better for this novel technique (27).

## Biocompatibility of Iron Oxide Nanoparticles

Iron is an essential element and, thus, a life without iron would not be possible. However, overload of iron is also hazardous since it can increase reactive oxygen species (ROS) production *via* Fenton and Haber-Weiss reactions (28). In the Fenton reaction (1), ferrous iron (Fe^2+^) reacts with hydrogen peroxide (H_2_O_2_) – which is, for instance, produced as a side product in mitochondrial respiration (29) – forming ferric iron (Fe^3+^), a hydroxide anion (OH^−^) and the extremely reactive hydroxyl radical (OH·). The oxidized Fe^3+^ can be reduced to Fe^2+^ again by the involvement of a superoxide anion, which will be converted to oxygen (2). The scheme below depicts the reaction equations of both reactions. The concluding final reaction is termed the Haber Weiss reaction – an iron catalyzed reaction, which generates hydroxyl radicals using hydrogen peroxide and superoxide radicals as substrates (3).

(1) $$\text{F}{\text{e}}^{2+}+{\text{H}}_{2}{\text{O}}_{2}\overset{}{\to }\text{F}{\text{e}}^{3+}+\text{O}{\text{H}}^{-}+\text{OH}\cdot$$

(2) $$\text{F}{\text{e}}^{3+}+{\text{O}}_{2}^{-}\cdot \overset{}{\to }\text{F}{\text{e}}^{2+}+{\text{O}}_{2}$$

(3) $$\;{\text{H}}_{2}{\text{O}}_{2}+{\text{O}}_{2}^{-}\cdot \overset{}{\to }{\text{O}}_{2}+\text{O}{\text{H}}^{-}+\text{OH}\cdot$$

Due to this reactions, cells have to tightly regulate iron metabolism and have developed numerous pathways and antioxidative defense mechanisms to compete iron-induced stress such as detoxification of H_2_O_2_ by the enzyme catalase or by involvement of the antioxidant glutathione and the enzymes glutathione peroxidase and glutathione reductase or by storing excess of free intracellular iron in the iron storage protein ferritin (30–32). Nevertheless, if these different antioxidative mechanisms become overcharged and fail, OH· radicals can induce intracellular damage such as lipid peroxidation, protein oxidation and subsequent degradation and DNA damage, which will lead to cell death through a process called ferroptosis (33). IONPs can be responsible in Fenton reaction-mediated ROS generation either at the particle surface or by intracellular liberation of Fe^2+^/Fe^3+^ ions (34–36). Oxidative stress is frequently described as the main reason for IONP-mediated cytotoxicity as extensively reviewed earlier (7, 37, 38).

### Cellular and Molecular Effects Determined *In Vitro*

Cytotoxicity of NPs can be assessed *in vitro* by a number of different assays addressing different cellular markers. The most prominent assays are the MTT-assay and the WST-1 assay to address the cellular metabolic activity, the LDH assay to address the cell membrane integrity and the Neutral Red uptake assay to assess lysosomal integrity. In general, the cytotoxicity of IONPs is considered rather low, especially when comparing them with other metal/metal oxide NPs such as silver or copper oxide (39, 40) and only a limited number of studies exists that report toxicity at lower (<100µg/mL) concentrations (41, 42). Interestingly, when moving away from the field of human toxicology, IONPs become much more critical. For example, Zhu and colleagues found that IONPs in concentrations of 10 µg/mL and higher severely affected hatching rate and survival from zebrafish (*Danio rerio*) while García and co-workers published EC50 values of even less than 1 µg/mL for IONP-toxicity towards *Daphnia magna* (43, 44).

Aside from classical cytotoxicity studies, numerous studies exist that in greater depth investigate the cellular and molecular effects of IONP exposure towards different cell types. As already mentioned above, oxidative stress plays a major role for IONP-related effects. For example, increase of ROS production was found for the IONP formulation Endorem^®^ in human liver cells (45), for Endorem^®^, Resovist^®^ and VSOP C200 in C17.2 neural progenitor cells and PC12 rat pheochromocytoma cells (46) or Feraheme^®^ in human T cells (47). However, also studies exist that show only minor or no ROS production after incubation of human cells with IONPs for bioimaging. Müller and colleagues showed that Ferumoxtran-10 was not toxic to human macrophages, nor did it activate them to produce pro-inflammatory cytokines or induce ROS production (48) and Lindemann and co-workers showed that Ferucarbotran (Resovist^®^) did not lead to increased ROS production in head and neck squamous cancer cells (HNSCCs) despite they induced apoptosis (49).

**Figure 1:**
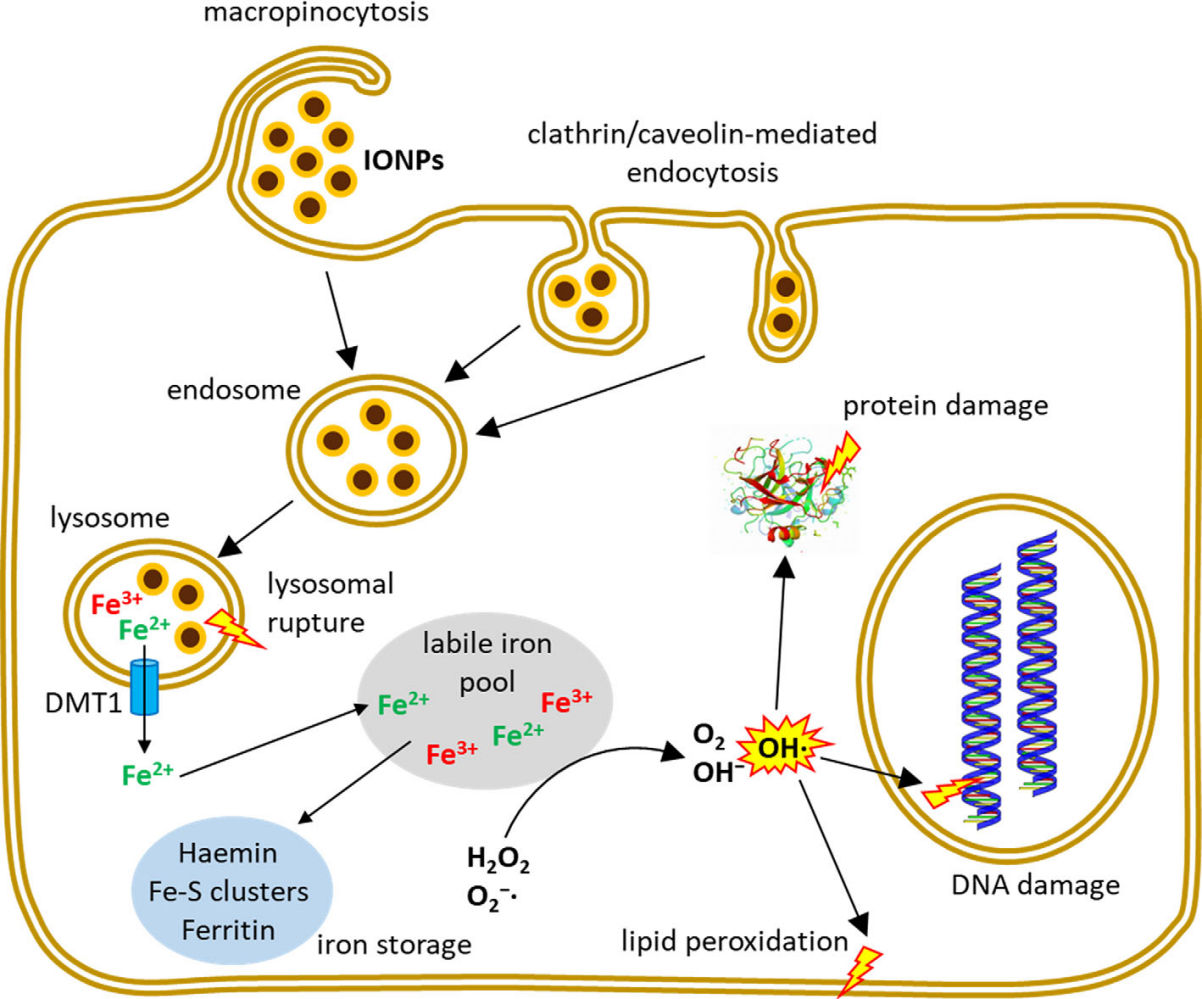
Uptake and intracellular fate of IONPs. Depending on IONP-size and coating and on the investigated cell type, different endocytic mechanisms contribute to the uptake of intact IONPs into the cells where the particles typically enter the endo-lysosomal system. Inside the cell, iron can be liberated from the NPs and contribute to several reactions including Fenton- and Haber-Weiss reactions leading to the formation of hydroxyl radicals which can induce cell damage.

In addition to ROS formation and cytotoxicity, other adverse effects have been reported being caused by IONPs. Dissanayake and co-workers extensively reviewed the potential of IONPs to induce genomic alterations (50). Genotoxicity of IONPs was observed by different types of DNA damage, such as chromosomal aberrations, DNA strand breakage, oxidative DNA damage and mutations (51) or by the formation of micronuclei (52). Jin and colleagues showed that the commercial IONP formulation Feridex^®^ exerted genetic toxicity in human hepatoma (HepG2) cells (53). Soenen and colleagues demonstrated that high concentrations of Endorem^®^, Resovist^®^ and citrate-coated IONPs induced important cytoskeleton and morphology alterations and affected the proliferation of C17.2 neural progenitor cells and primary human blood outgrowth endothelial cells (46, 54). Other IONP-related effects that have been reported are cell cycle alterations (55), autophagy (56) or apoptosis (49, 57). **Figure 1** summarizes entry routes and cellular fate of IONPs in human cells.

### Biodistribution, Clearance and Toxicity Determined *In Vivo*

Understanding the biodistribution of NPs is a critical point when evaluating their biological and immunological safety (58). The disposition of any compound within an organism can be described by the ADME principle, which stands for **a**bsorption, **d**istribution, **m**etabolism and **e**xcretion. For IONPs, all these four criteria are strongly dependent on particle characteristics, such as size, size distribution, surface charge, coating molecules and protein corona formation (59). Already more than 30 years ago, the biodistribution of radioactive-labelled Ferumoxide (AMI-25) after injection in rats was studied, with the result that the majority of the particles accumulated in the liver and spleen 1 h after injection (60). While the clearance of the particles of these organs occurs with half-lives of 3-4 days, the whole body clearance was much longer (44.9 days). In contrast, the blood half-life of these particles was determined to be only 6 min, indicating the rapid uptake and clearance by the mononuclear phagocyte system (MPS) (61). In the same report, the authors also investigated the blood half-life of a second ultrasmall IONP type (size <10 nm, while AMI-25 was 72 nm) and found that these particles remain in the bloodstream with half-lives of 81 min, indicating that IONP size plays a crucial role in particle clearance. This finding was also confirmed by Bourrinet and colleagues who compared 30 nm Ferumoxtran-10 with the larger Ferumoxides (62).

Apart from IONP size, also the coating and surface charge play a major role in their blood clearance, pharmacokinetics and biodistribution (58, 59). In general, IONPs with neutral surface charge are considered having a longer circulation time and a reduced uptake by the MPS due to less opsonization (63, 64). Polyethylene glycol (PEG) is often used as a coating agent for IONPs, since it provides a steric stabilization of the particles by a shielding of their surface charge that strongly reduces opsonization and subsequent macrophage uptake (65). Another polymeric coating agent, which is often used as alternative to PEG, is polyvinyl pyrrolidone (PVP) (66). IONPs with positively charged coatings are often cleared much faster from the bloodstream due to differences in protein absorption in comparison with neutral or negatively charged particles (67). It is well known that protein absorption and subsequent protein corona formation has a great impact on the biological identity of NPs and on their effects on living organisms (68). When cleared from the bloodstream, injected IONPs are mainly found in the liver and spleen. However, there is also evidence of IONPs present in the lungs (69), kidney (70), heart (71) and even the brain (72).

IONPs are generally considered as safe and non-toxic *in vivo* (59), however, such particles can have side effects such as local pain, hypotension, hypersensitivity, anaphylactic shock, vasodilatation and paraesthesia (9) which were the reasons for withdrawal of the earlier clinical applied dextran/carboxydextran-coated IONP-formulations (Feridex^®^/Endorem^®^, Resovist^®^/Cliavist^®^, Sinerem^®^/Combidex^®^). A still approved formulation is Ferumoxytol (Feraheme^®^, Rienso^®^ in the EU) which is primarily used as iron therapy and has been shown to be tolerable in high doses up to 510 mg per injection (73). However, there is also evidence of risks of hypersensitivity reactions and also the long-term effects seem to be not fully evaluated (74).

## Immune Effects of Iron Oxide Nanoparticles

Besides cytotoxicity induced through particle-mediated or iron-mediated oxidative stress, IONPs may be recognized by the immune system and can, thus, induce different immunological effects. These immune effects are – just like the aforementioned cellular and molecular effects – highly dependent on the particle characteristics. Size, shape, surface charge and particle coating have been described previously to greatly influence the immune effects of IONPs (9). On the one hand, this is explained by the fact that these parameters also influence the IONP biodistribution and toxicokinetic profile (75); one the other hand, certain coatings have been shown to induce certain immune effects. For example, polyethyleneimine (PEI)-coated IONPs have been shown to enhance Th1 polarization of human dendritic cells (DCs) (76), while dextran-coated IONPs have been shown to suppress the proliferation activity of T-lymphocytes (77).

As already mentioned in the previous sections, the first reaction of the human immune system on injected IONPs will be their rapid uptake and elimination by cells of the MPS. One essential component of the MPS are monocytes, which circulate in the peripheral blood and can become activated by different stimuli. Earlier studies have shown that monocytes accumulate dextran-coated IONPs and that this accumulation induces autophagy and an increase in secretion of the pro-inflammatory cytokines Interleukin (IL)-1β, IL-6 and Tumor Necrosis Factor (TNF)-α (78). Contradictory results have been published by Grosse and co-workers who showed that 10 and 30 nm IONPs (coated with a monolayer of oleic acid and a monolayer of amphiphilic polymer) did not lead to elevation of these cytokines end even inhibited LPS-induced pro-inflammatory cytokine production (79). A likely reason for this discrepancy could be the fact that in the latter study, the particles were proven to be free of LPS or other TLR4 agonists, while the authors of the first study did not investigate this. Moreover, the different coatings of the particles can play a role in their different immunological responses. For example, amino-functionalized amino-polyvinyl alcohol-coated IONPs have been shown to increase IL-1β production of human monocytes (80), while starch-coated IONPs did not alter IL-1β and IL-10 secretion but downregulated IL-6 secretion in primary monocytes (81). These differences in observed cytokine profiles shows the need for future studies in order to gain a mechanistic understanding of the stimulatory or suppressive effects of IONPs towards human monocytes.

Macrophages are phagocytes, which can be activated in two different ways: classical (M1) or alternative (M2). The main function of M1 macrophages is the detection, phagocytosis and killing of pathogens, apoptotic cells and damaged host cells while M2 macrophages have important functions in tissue repair (9). Aside from this, macrophages can function as antigen-presenting cells (APCs) and thereby connect innate with adaptive immunity. IONPs have been frequently used to label macrophages for MRI, for example in the brain (82), and have been shown to perform better than conventional Gd-based contrast agents in a model of multiple sclerosis (83). On the other hand, it has been shown *in vitro* that IONPs can also induce oxidative stress and affect cell viability of rat microglia cells (36). Other mechanistic studies reported that IONPs lead to a secretion of IL-12, IL-1β and to upregulation of several genes linked to the M1 phenotype in murine and human macrophages (80, 84, 85). However, it should be noted here that the IONPs in these studies were not checked for endotoxin contaminations. In a study by Yang and colleagues, the clinically applied IONP formulation Ferucarbotran has been shown to activate RAW264.7 macrophages by an increase in oxidative stress, a decrease in mitochondrial membrane potential and an increase in cell proliferation (86).

IONPs can also affect the function of APCs as shown in a study by Park and co-workers (87). They exposed mice intratracheally with IONPs and found that these particles remained in the lungs for at least 90 days and enhanced the expression of antigen presentation-related proteins such as CD80, CD86, and MHC class II, on APCs in bronchoalveolar lavage (BAL) fluid. Another study by Mou and colleagues reported that positively charged IONPs enhanced antigen cross-presentation of murine DCs while negatively charged IONPs inhibited the DCs’ functions and rapidly activated autophagy (88). The clinical IONP-formulation Ferumoxide (Feridex^®^/Endorem^®^) had been shown earlier to affect the viability and migratory properties of DCs, however, only at high concentrations (89, 90).

The principal cells of humoral and cellular immune response are the lymphocytes, which specifically recognize and respond to antigens and produce antibodies (B cells) or directly kill infected cells or assist the MPS in destroying them (T cells). The ability of these cells to take up NPs is, generally, considered lower than that of monocytes or macrophages (9). A study by Gaharwar and colleagues reports that isolated rat splenic lymphocytes take up IONPs and that the particles affect cell viability, increase intracellular ROS levels and lipid peroxidation while depleting antioxidative enzymes and glutathione (91). Resovist^®^ has been shown to attenuate Th17 responses in ovalbumin (OVA)-sensitized BALB/c mice *in vivo* and in OVA-primed splenocytes isolated from BALB/c mice *in vitro* (92). This was indicated by a decreased infiltration of CCR6+, IL-6+, IL17+ and ROR-γ+ cells in inflamed footpads *in vivo* and by a suppression of the expression of IL-6, IL-17 and ROR-γt *in vitro*. In another study, Feraheme^®^ has been shown to suppress the immune function of human T lymphocytes through mitochondrial damage and ROS production (47). In contrast, a Th1-type immune activation was described by Zhu and co-workers, who exposed mice intratracheally with 4 or 20 µg IONPs and reported that NP-induced exosomes were responsible as signaling mediators for this Th1 immune activation (93).

Taken together, it can be stated that there are a number of open questions regarding the complete understanding of IONPs on the functioning of different immune cells. **Table 2** sums up the IONP-derived immune effects and the cytokines involved of the studies on different immune cells reviewed in this paper. Results presented in literature are sometimes inconsistent which can be attributed to the use of different IONPs, IONP-coatings, -sizes or -shapes or due to different incubation conditions. Structure-activity relationships are not always clear and future studies are definitely needed in order to systematically evaluate the immune effects of IONPs and the influence of different particle characteristics (9).

**Table 2:** IONP-derived immune effects and up-/downregulated cytokines of the studies reviewed in this articles.

Cell type	Immune effects / cytokines up- or downregulated	Reference
Monocytes	Autophagy / IL-1β (+), IL-6 (+), TNFα (+)	(55)
	Suppression of LPS-induced NFκB activation	(79)
	IL-1β (+)	(80)
	IL-6 (–)	(81)
Macrophages	M1 activation, IL-12 (+)	(84)
APCs	IL-1β (+)	(85)
DCs	Upregulation of several proinflammatory cytokines	(80)
Lymphocytes	expression of CD80, CD86 and MHC class II	(87)
	autophagy / antigen cross-presentation	(88)
	oxidative stress, glutathione depletion	(91)
	Attenuation of Th17 cell responses	(91)
	IL-6 (–), IL-17 (–), ROR-γt (–)	(92)
	Suppression of T-lymphocytes	(47)
	Th1 immune activation	(93)

## Conclusions and Future Perspectives

Iron oxide nanoparticles (IONPs) possess a variety of biomedical applications, especially in bioimaging. However, these applications came with a price since many of the IONP-containing formulations have shown to cause different side-effects that later resulted in their withdrawal from the market. We herein reviewed the biological and immunological effects of IONPs and concluded that published results often show inconsistencies regarding the effects of such particles. Theses inconsistencies can often be attributed to the particle characteristics (size, shape, surface coating) or the experimental design and incubation conditions (*e.g.*, the presence of bystander substances such as bacterial endotoxins). Thus, quantitative structure-activity relationships (QSARs) for the analysis of such particles are difficult to draw and require more data from future studies involving systematic analysis of well-characterized particles under clearly defined experimental conditions.

## Author Contributions

MG performed literature search and prepared first draft and display items of mini review. MH was involved in critical discussions of the content and display items, writing and editing of the mini review. All authors contributed to the article and approved the submitted version.

## Funding

This work was funded by the H2020 EU research infrastructure for nanosafety projects NanoCommons (Grant Agreement No. 731032) and NanoRigo (Grant Agreement No. 814530) and by the Allergy-Cancer-BioNano Research Center of the Paris Lodron University of Salzburg.

## Conflict of Interest

The authors declare that the research was conducted in the absence of any commercial or financial relationships that could be construed as a potential conflict of interest.
